# Dual Epigenetic Regulation of ERα36 Expression in Breast Cancer Cells

**DOI:** 10.3390/ijms20112637

**Published:** 2019-05-29

**Authors:** Charlène Thiebaut, Amand Chesnel, Jean-Louis Merlin, Maelle Chesnel, Agnès Leroux, Alexandre Harlé, Hélène Dumond

**Affiliations:** 1 Université de Lorraine, CNRS, CRAN, F-54000 Nancy, France; charlene.thiebaut@univ-lorraine.fr (C.T.); amand.chesnel@univ-lorraine.fr (A.C.); maelle.chesnel22@gmail.com (M.C.); 2 Université de Lorraine, CNRS, CRAN, Institut de Cancérologie de Lorraine, F-54000 Nancy, France; jl.merlin@nancy.unicancer.fr (J.-L.M.); alexandre.harle@crick.ac.uk (A.H.); 3 Institut de Cancérologie de Lorraine, F-54000 Nancy, France; a.leroux@nancy.unicancer.fr

**Keywords:** breast cancer, ERα36, microRNA, methylation, endocrine therapy resistance

## Abstract

Breast cancer remains the major cause of cancer-induced morbidity and mortality in women. Among the different molecular subtypes, luminal tumors yet considered of good prognosis often develop acquired resistance to endocrine therapy. Recently, misregulation of ERα36 was reported to play a crucial role in this process. High expression of this ERα isoform was associated to preneoplastic phenotype in mammary epithelial cells, disease progression, and enhanced resistance to therapeutic agents in breast tumors. In this study, we identified two mechanisms that could together contribute to ERα36 expression regulation. We first focused on hsa-miR-136-5p, an ERα36 3’UTR-targeting microRNA, the expression of which inversely correlated to the ERα36 one in breast cancer cells. Transfection of hsa-miR136-5p mimic in MCF-7 cells resulted in downregulation of ERα36. Moreover, the demethylating agent decitabine was able to stimulate hsa-miR-136-5p endogenous expression, thus indirectly decreasing ERα36 expression and counteracting tamoxifen-dependent stimulation. The methylation status of ERα36 promoter also directly modulated its expression level, as demonstrated after decitabine treatment of breast cancer cell and confirmed in a set of tumor samples. Taken together, these results open the way to a direct and an indirect ERα36 epigenetic modulation by decitabine as a promising clinical strategy to counteract acquired resistance to treatment and prevent relapse.

## 1. Introduction

Despite considerable improvement of therapeutic strategies, breast cancer remains a leading cause of cancer mortality in women, mainly due to acquired resistance to treatment. Indeed, 30–50% of luminal tumors, yet considered as highly curable, develop endocrine therapy resistance through unclear and diverse mechanisms, including alterations of estrogen receptor (ER) gene sequence or epigenetic marks, alternative signaling pathway usage or variation in the pattern of ER isoform [1]. Recently, Wang and colleagues argued that one of the key actors of such acquired resistance could be the ER variant ERα36, the expression and/or activity of which become stimulated under estrogen deprivation or low dose tamoxifen exposure [2,3,4,5].

ERα36 is a 36kDa receptor encoded by the ESR1 locus and transcribed from an alternative promoter located into the first intron [6]. Compared to the canonical ERα66 protein, alternative splicing results in a shortened receptor lacking both AF-1 and AF-2 transactivation domains as well as the C-terminal part of the ligand-binding domain. ERα36 retains dimerization and DNA-binding domains and contains a unique 27 amino acid C-terminal sequence involved in cofactors interactions [7,8]. 17β-estradiol (E2) and hydroxy-tamoxifen (OHT) are able to activate ERα36, which is primarily located below the cell surface and triggers nongenomic pathway stimulation (JAK/STAT, PI3K/AKT, MEK/ERK) [9]. ERα36 overexpression in mouse normal mammary epithelium is related to several phenotypic hallmarks of breast cancer cells, such as loss of adhesion and enhanced migration potential, resistance to apoptosis, and genetic instability [10]. Forced or endogenously high expression of ERα36 in breast cancer cell lines or tumor samples is also known to stimulate proliferation, migration, stemness, and resistance to therapeutic agents, independently of the ERα66 protein level [2], [11,12,13]. 

Classic regulation of ERα36 expression by transacting factors has previously been investigated in breast cancer cells: ERα66 was described as a negative regulator [14], whereas AP-1 family proteins, EGFR and HER2 as positive ones [15]. An epigenetic regulation of ERα36 promoter activity was also suggested after mammary epithelial cell exposure to a long chain alkylphenol mixture [16]. In addition, accumulative data point out the role of microRNAs in the regulation of oncogenes and tumor suppressors [17]. Among the myriad of microRNAs identified in regulating oncogenesis, hsa-miR-136-5p could be a good candidate for ERα36 regulation. Indeed, miR-136 expression negatively correlates to the tumor stage and metastatic potential in breast cell lines and tumor biopsies: hsa-miR-136-5p expression is high in normal tissue and MCF-10A cells where ERα36 is absent, moderate in WHO stage I and II samples and luminal MCF-7 cells where ERα36 expression level is low, and low in high grade tumors and triple-negative breast cancer cell (TNBC) lines such as MDA-MB-231 in which ERα36 level is high [3,18,19]. Additionally, hsa-miR-136-5p is able to target RASAL2 in TNBC and subsequently suppresses migration and invasion, two processes which may involve ERα36 functionality [18]. More generally, hsa-miR-136-5p is described as a tumor suppressor in many solid tumor models such as breast, prostate, ovarian, cervical, colon, glioma, osteosarcoma, and lung squamous cell cancer. Depending on the organ, it targets key actors of kinase-dependent transduction pathways (RASAL2, TCFPC2, MAP2K4, PTEN, wnt2) or downstream effectors (Bcl2, MMP3, MIEN1, metadherin, etc.) and thus participates to the control of proliferation, migration, invasion, and apoptosis [18], [20,21,22,23,24]. 

In an attempt to identify modulators of ERα36 expression, we first screened microRNAs specifically targeting ERα36. We selected 12 microRNAs and focused on hsa-miR-136-5p because (i) its expression in breast cancer cells appeared inversely correlated to ERα36, (ii) both ERα36 and hsa-miR-136-5p were involved in the same cancer cell features, and (iii) hsa-miR-136-5p was predicted to bind ERα36 3’UTR. Moreover, we showed that the demethylating agent decitabine (DAC) modulated ERα36 expression, directly by targeting the ERα36 promoter region and indirectly by stimulating hsa-miR-136-5p expression, thus counteracting tamoxifen-induced ERα36 overexpression. 

## 2. Results

### 2.1. Screening of microRNAs that Target ERα36 but not ERα66

**Figure 1:**
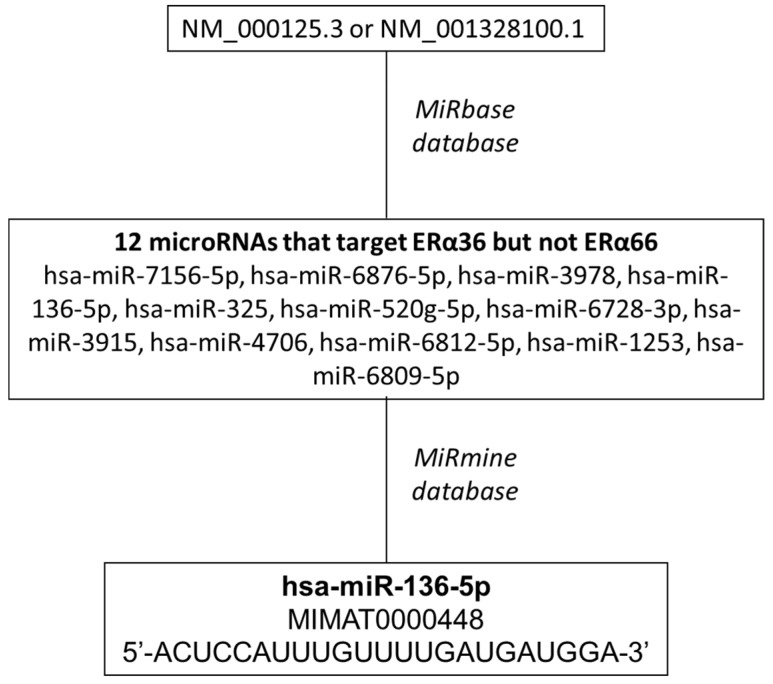
Strategy for the screening of microRNAs that target ERα36 but not ERα66.

In a first step, we screened MiRbase database for microRNAs that target Homo sapiens estrogen receptor 1 (ESR1), transcript variant 7, 3’UTR (Acc number: NM_001328100.1) encoding ERα36 protein but not Homo sapiens estrogen receptor 1 (ESR1), transcript variant 1, 3’UTR (Acc number: NM_000125.3) encoding ERα66 (Figure 1) [25,26,27,28]. Twelve microRNAs predicted to potentially hybridize specifically to the 3’UTR of ERα36 encoding transcript: hsa-miR-7156-5p, hsa-miR-6876-5p, hsa-miR-3978, hsa-miR-136-5p, hsa-miR-325, hsa-miR-520g-5p, hsa-miR-6728-3p, hsa-miR-3915, hsa-miR-4706, hsa-miR-6812-5p, hsa-miR-1253, hsa-miR-6809-5p. Each microRNA was submitted to the miRmine database to check its expression in normal/tumor breast tissue or cell lines. hsa-miR-136-5p (mature_acc = MIMAT0000448; 5’-ACUCCAUUUGUUUUGAUGAUGGA-3’) displayed the highest expression level among the 12 candidates in MCF-7 breast cancer cells and was found two times less in sera from breast tumor patients than from normal ones [29].

### 2.2. hsa-miR-136-5p Under/over Expression Modulates ERα36 mRNA Expression Level

**Figure 2:**
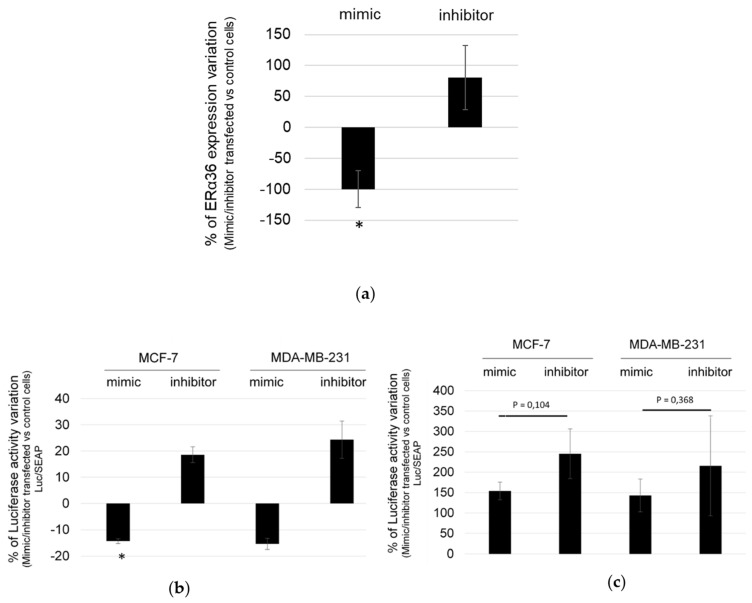
hsa-miR-136-5p mimic/inhibitor directly modulates ERα36 expression. (**a**) MCF-7 cells were transfected with either hsa-miR-136-5p mimic or inhibitor. ERa36 expression was measured by RT-qPCR analysis 24 h after transfection, and the percentage of ERα36 expression variation in hsa-miR-136-5p mimic or inhibitor transfected cells versus control ones was calculated. MCF-7 or MDA-MB-231 cells were co-transfected with hsa-miR-136-5p mimic or inhibitor and (**b**) the pMIR-Report-Luc-ERα36wt plasmid or (**c**) the pMIR-Report-Luc-ERα36mut one. Luciferase activity was measured 24 h after transfection using the Bright-Glo™ luciferase assay system. To evaluate the transfection efficacy and normalize the results, cells were also transfected at the same time with the pEZX-PG04-Secreted alkaline phosphatase plasmid. The alkaline phosphatase activity was measured 24 h after transfection in the culture medium with the Secrete-Pair™ Gaussia luciferase dual and single luminescence assay Kit. Each bar represents mean ± S.E.M. (a) *N* = 5 (b) *N* = 3 and (c) *N* = 5. * *P* < 0.05.

To confirm that hsa-miR-136-5p modulate ERα36 expression, MCF-7 cells were transfected with either hsa-miR-136-5p mimic or inhibitor. ERα36 mRNA and hsa-miR-136-5p expression levels were measured by RT-qPCR analysis. The ratio of ERα36 expression in hsa-miR-136-5p mimic- or inhibitor-transfected versus control cells indicated that ERα36 mRNA expression level significantly decreased by 100% in the case of hsa-miR-136-5p mimic transfection compared to the hsa-miR-136-5p inhibitor one (Figure 2A). Transfection with hsa-miR-136-5p inhibitor led to an enhanced ERα36 expression (Figure 2A), but this tendency remained not statistically significant, probably due to experimental variation in inhibitor transfection efficacy (data not shown).

The specific interaction between hsa-miR-136-5p and the 3’UTR region of ERα36 mRNA was then assessed in both MCF-7 and MDA-MB-231 cells, which both express ERα36 but belong either to luminal A or to triple-negative molecular subtype, respectively (Figure S1). The plasmid pMIR-Report-Luc-ERα36wt (wt) contained the wild-type hsa-miR-136-5p target sequence of ERα36 3’UTR and pMIR-Report-Luc-ERα36mut (mut), a corresponding mutated sequence. Both the wt and mut sequence were cloned downstream the luciferase gene, thus driving luciferase mRNA stability in the presence of hsa-miR-136-5p. The cells were co-transfected with either the wt or the mut plasmid and hsa-miR-136-5p mimic or inhibitor. Luciferase activity was measured 24 h after transfection and normalized for transfection efficacy using the alkaline phosphatase activity from the co-transfected pEZX-PG04-Secreted alkaline phosphatase plasmid. Transfection of hsa-miR-136-5p mimic or inhibitor in MCF-7 or MDA-MB-231 cells resulted in a 120% to 150% decrease and a 180% to 240% increase of luciferase activity compared to control cells, respectively (Figure 2B). Both cell lines transfected by the mut plasmid displayed a higher basal level of luciferase activity than the wt one. There was no statistical variation between the hsa-miR-136-5p mimic or inhibitor transfected cells (Figure 2C). Therefore, we concluded that hsa-miR-136-5p directly and specifically targeted ERα36 mRNA, thus modulating its expression level in breast cancer cells.

### 2.3. The Demethylating Agent DAC Stimulates hsa-miR-136-5p and Represses ERα36 Expression

**Figure 3:**
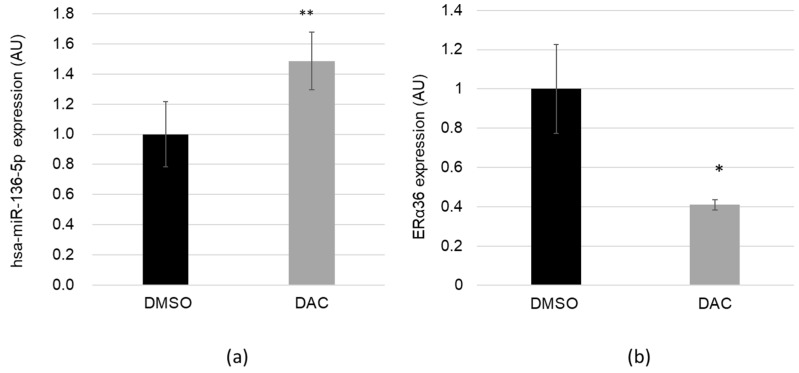
The demethylating agent decitabine (DAC) stimulates hsa-miR-136-5p expression and represses ERα36 one. MCF-7 cells were treated for 48 h with DMSO 0.01% (as control) or the demethylating agent DAC 5 µM. (**a**) hsa-miR-136-5p expression level was assessed using TaqMan™ microRNA assay and (**b**) ERα36 expression was measured by RT-qPCR analysis. Each bar represents mean ± S.E.M. (a) *N* = 6 (b) *N* ≥ 3. * *P* < 0.05; ** *P* < 0.01.

Many microRNAs are epigenetically regulated trough the methylation status of their promoter [30]. hsa-miR-136-5p is located in the DLK1-DIO3 imprinted locus of chromosome 14q32, which contains a cluster of noncoding RNAs and the antisense gene RTL1, all differentially regulated by the methylation status of the IG-DMR. Methylation changes in this locus, which may affect gene or microRNA expression associated with Temple or Kagami–Ogata syndrome and oligometastastic phenotype [31,32]. Therefore, we hypothesized that expression of hsa-miR-136-5p could be upregulated after cell exposure to the demethylating agent DAC and thus downregulate ERα36. The MCF-7 cells were treated for 48 h with DAC or DMSO as control. DAC exposure did not alter cell viability (data not shown). MicroRNAs and long mRNAs were harvested from the same cells. hsa-miR-136-5p expression level (Figure 3A) and ERα36 expression (Figure 3B) were measured using RT-qPCR. DAC treatment induced a significant 48% increase of hsa-miR-136-5p expression as well as a 59% decrease of ERα36 expression. The upregulation was independent of the hsa-miR-136-5p sponge CRNDE (colorectal neoplasia differentially expressed gene) (Figure S2).

### 2.4. DAC Counteracts OHT-dependent Stimulation of ERα36 Expression

**Figure 4:**
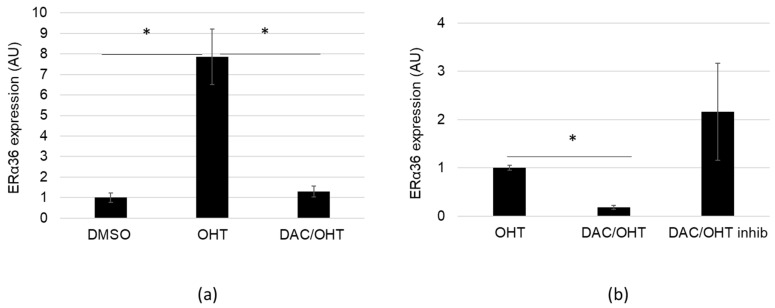
Decitabine (DAC) treatment counteracts 4-hydroxy-tamoxifen (OHT) induced ERα36 expression. (**a**) MCF-7 cells were treated for 48 h with DMSO (as control), OHT 1 µM or by a combination OHT 1 µM/DAC 5 µM. ERα36 expression was measured by RT-qPCR analysis. (**b**) MCF-7 cells were transfected or not with hsa-miR-136-5p inhibitor. Both control and hsa-miR-136-5p inhibitor transfected cells were treated for 48 h by a combination of OHT 1 µM/DAC 5 µM. ERα36 expression was measured by RT-qPCR analysis. OHT treatment was used as reference. Each bar represents mean ± S.E.M. *N* ≥ 3. * *P* < 0.05.

OHT was previously reported to either stimulate or repress ERα36 expression in MCF-7 cells exposed to 1 µM or 5 µM, respectively [5,33]. Since DAC (5 µM) and OHT (1 µM) displayed inverse effects on ERα36 expression, MCF-7 cells were exposed either to DAC or to OHT (1 µM), or co-treated with both compounds for 48 h. Exposure to 1 µM OHT-induced ERα36 expression by 8-fold although hsa-miR-136-5p expression was not affected by the treatment (data not shown). Moreover, co-treatment with DAC and OHT was able to counteract OHT-dependent stimulation of ERα36 expression (Figure 4A). Transfection of DAC/OHT co-treated cells with hsa-miR-136-5p inhibitor seemed to cancel the DAC-dependent impairment of OHT effect (Figure 4B), indicating that hsa-miR-136-5p was indeed a key element in this process. The variability of ERα36 expression observed in the [DAC/OHT + hsa-miR-136-5p inhibitor] condition was most likely due to uncontrolled experimental variations of the hsa-miR-136-5p inhibitor amount present into the cells. Taken together, these data suggested that DAC could be used to chemically repress ERα36 expression and prevent its induction by OHT through the stimulation of hsa-miR-136-5p.

### 2.5. ERα36 Expression May be Modulated by the Methylation Status of Its Promoter, In Vivo and In Vitro

**Figure 5:**
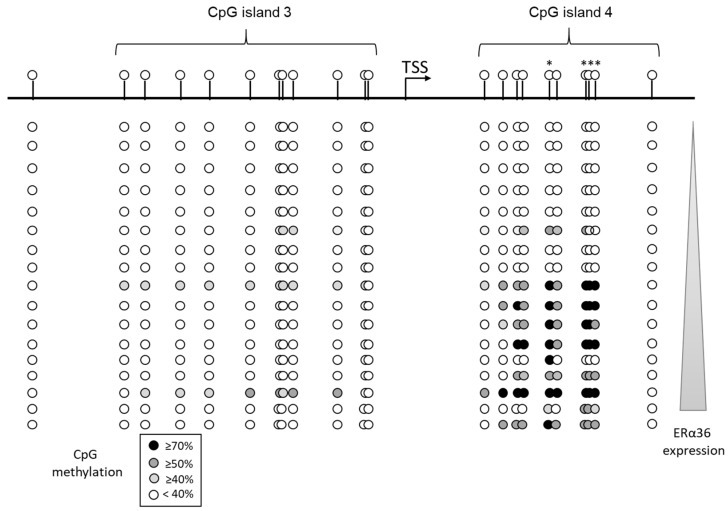
ERα36 expression is related to the methylation status of its promoter in breast tumor samples. Seventeen breast tumor samples were collected from the tumor biobank of the Cancerology Institute of Lorraine. ERα36 promoter genomic DNA was submitted to bisulfite sequencing and ERα36 expression level was performed by real-time PCR analyses in triplicates for each tumor sample. The correlation between each CpG methylation and ERα36 expression was established by a MATLAB program (Pearson rank correlation test). * CpG which methylation level is significantly (*P* < 0.05) correlated to ERα36 expression level.

Previous studies from our laboratory [16,34] suggested that ERα36 expression may be modulated by the methylation status of its promoter sequence, as defined by Wang’s laboratory [14]. We searched for CpG islands located in the first intron of ESR1 using the EMBOSS CpG plot website (http://www.ebi.ac.uk/Tools/seqstats/emboss_cpgplot/) and identified four clusters (Figure S3). The methylation status of corresponding sequences was determined after bisulfite conversion in a subset of 17 breast primary tumors collected from the biobank of the Cancerology Institute of Lorraine (Vandoeuvre-lès-Nancy, France). Total RNAs were harvested from the same samples in order to measure ERα36 expression level by RT-qPCR. The correlation between each CpG methylation level and ERα36 expression level was addressed via a Pearson rank statistical test (Figure 5). The results clearly suggested that the methylation of 4 CpGs located on the island 4 was correlated to a high ERα36 expression.

### 2.6. DAC Directly Targets ERα36 Genomic Sequence

**Figure 6:**
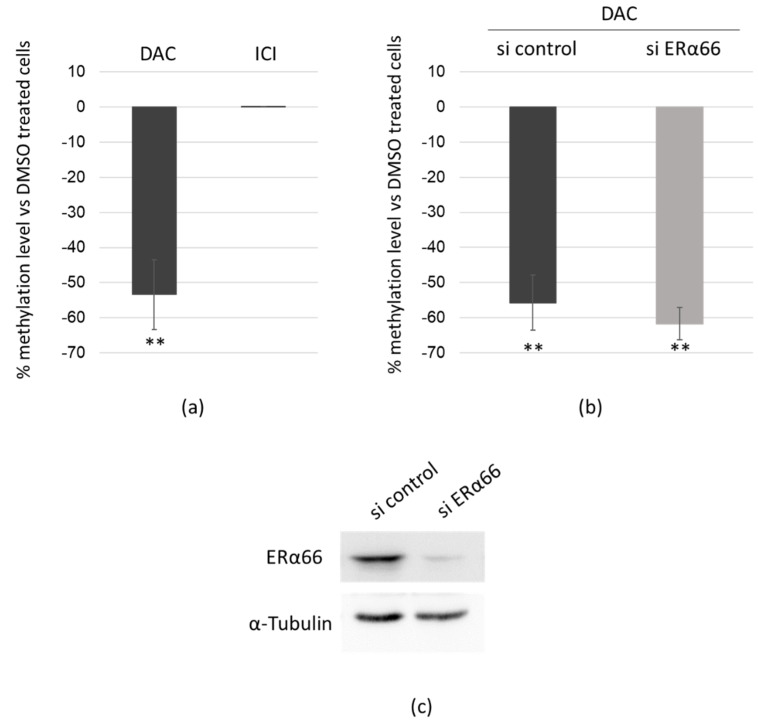
Decitabine directly targets ERα36 genomic sequence. (**a**) MCF-7 cells were treated for 48 h with DMSO (as control), DAC or ICI 182,780 (*N* = 4); (**b**) MCF-7 cells transfected with either scrambled siRNA (si control, *N* = 4) or siRNA targeting the exon 1 of ESR1 (siERα66, *N* = 3) were treated for 48 h with DMSO (as control) or DAC. The methylation level of the ERα36 promoter fourth CpG island was assessed after bisulfite conversion. A percentage of methylation level variation was calculated for DAC or ICI treated cells versus DMSO ones. (**c**) Representative Western blot analysis of ERα66 expression in MCF-7 cells transfected with either si control or si ERα66. α-Tubulin was used as a loading control. Each bar represents mean ± S.E.M. ** *P* < 0.01.

Since the methylation status of ERα36 promoter seemed to be linked to its expression in tumor biopsies, we addressed a direct effect of the demethylating agent DAC on ERα36 expression through demethylation of its promoter. MCF-7 cells were exposed to DAC for 48 h, and the methylation status of the four CpGs significantly related to ERα36 expression was assessed after bisulfite conversion by real-time PCR. ERα66 is known to repress ERα36 expression and was reported to regulate its target genes through epigenetic modulator recruitment [35]. Thus, we compared the effect of the exposure to DAC or to the ERα66 inhibitor fulvestrant (Figure 6A). DAC triggered a 53% decrease in the methylation level of the ERα36 promoter sequence where ICI treatment had no effect. The same experiment was performed in MCF-7 cells transfected by an ERα66 siRNA to knockdown ERα66 and exposed to DAC for 48 h (Figure 6B). The ERα66 knockdown was confirmed by Western blot (Figure 6C). DAC significantly and similarly demethylated ERα36 promoter in both sicontrol and siERα66 transfected cells. This confirmed that DAC directly acted on ERα36 promoter sequence independently of ERα66 and further suggested that the DAC-dependent repression of ERα36 expression could account for (i) an indirect effect through the hsa-miR-136-5p targeting of ERα36 3’UTR and (ii) a direct effect through demethylation of its promoter.

## 3. Discussion

This study depicts, for the first time, two epigenetic mechanisms that regulate ERα36 expression in breast cancer cells. In line with the study of Li and colleagues [5], we showed that ERα36 expression increased significantly after a low dose tamoxifen exposure in MCF-7 cell line. This upregulation was previously associated with a decreased sensitivity to tamoxifen and enhanced proliferative, migratory, and invasive abilities of breast cancer cells [5,15]. Mechanistically, computational modeling and docking analysis revealed that tamoxifen is able to bind directly ERα36 through its ligand-binding domain [2]. This interaction activated various estrogen nongenomic pathways such as MAPK/Erk and PI3K/Akt as well as the ERα36/EGFR/HER2 regulatory loop responsible for tamoxifen therapy escape [9,15,36]. These experimental results provided support and explanations to clinical studies wherein a high ERα36 expression in tumors treated with tamoxifen was significantly associated with a poor prognosis and an increased rate of metastases [2,37]. In this context, the understanding of mechanisms that regulate ERα36 expression was important to try preventing the deleterious effect of ERα36-triggered response to tamoxifen. 

First, we showed in vitro and in breast tumor samples that a low methylation level of 4 CpGs localized in the promoter of ERα36 correlated with a low ERα36 mRNA expression. A demethylation of this CpG rich region was namely obtained in vitro after a 48 h DAC exposure which led to ERα36 downregulation. This supported the causal relationship between the methylation status of the promoter, and the expression level of ERα36. ERα66 has been described as the main direct transcriptional regulator of ERα36 expression and is able to regulate his target genes through the methylation level of their promoter [14,35,38,39]. When we silenced ERα66 expression or activity by either siRNAs or ICI 280 182, we showed that ERα66 did not drive the methylation-dependent repression of ERα36. 

DAC is an FDA-approved hypomethylating agent, currently used to treat blood cancer. Recently, a study conducted by researchers from Florida on animal models demonstrated that a low dose of DAC prevented breast cancer cells from spreading and could also be used to treat aggressive chemoresistant triple-negative breast cancer [40,41]. As previously described in the case of ERβ silencing [42], treatment with DAC may promote ERα36 promoter demethylation and thus override the molecular mechanisms underlying ERα36-dependent tamoxifen resistance. 

Second, our data indicated that DAC also acted indirectly on ERα36 expression since it upregulated hsa-miR-136-5p expression which, in turn, targeted ERα36 mRNA. hsa-miR-136-5p was described as a tumor suppressor microRNA, associated with impaired tumorigenesis and metastasis in prostate cancer by targeting MAP2K4 [43], renal cell carcinoma [23], lung squamous cell carcinoma by controlling metabolic processes [22], lung adenocarcinoma by suppressing cell adhesion gene expression [24], and glioma and cutaneous squamous cell carcinoma by targeting WNT signaling [44,45]. The downregulation of hsa-miR-136-5p has been described in a chemo-radioresistant group of patients with advanced stage cervical squamous cell carcinoma. Conversely, a high ERα36 expression was associated to estrogen nongenomic signaling driving metastatic progression, acquired resistance to hormone treatment, and poor outcome [11,13,46].

Our in vitro data demonstrated that hsa-miR-136-5p directly targeted ERα36 3’UTR and led to mRNA expression downregulation. These data suggested that the upregulation of ERα36 expression in response to OHT could be impaired either by the direct mimicking or by the DAC-dependent indirect stimulation of hsa-miR-136-5p that may consequently override ERα36-dependent resistance to OHT. Further in vivo experiments are needed to explore whether treatment with DAC, prior or in combination with OHT, would be a relevant therapeutic strategy.

In the mammary gland, hsa-miR-136-5p expression was higher in normal breast tissue than in breast carcinoma and in luminal vs. triple-negative breast cancer cell lines [18,19,47]. In line, Pascek and coworkers described a significant higher expression in luminal tumors than in TNBCs [48]. However, other studies from tumor biopsies did not report hsa-miR-136-5p expression as a reliable marker for predicting either breast cancer molecular type [49], pathological stage [50] or patient outcome [51]. Little is known about hsa-miR-136-5p regulation by DNA methylation status. The promoter sequence of hsa-miR-136-5p has not been clearly defined. A 135 bp sequence is located upstream hsa-miR-136-5p and separates hsa-miR-437-5p from hsa-miR-136-5p, but the promoter status of this sequence or the relationship between its methylation level and hsa-miR-136-5p expression are not described. Moreover, hsa-miR-136-5p is located in the 14q32.2 Dlk1-Dio3 chromosomal region, which has been described to be submitted to global and complex regulation by parental imprinting through two DMR [52]. Therefore, it would be interesting to determine precisely the regulatory elements of hsa-miR-136-5p and to set up a prospective study in order to investigate both its expression and the methylation status of its putative promoter in breast tumors. 

Expression of hsa-miR-136-5p was also tightly regulated by a combination of sponge lncRNAs, such as CRNDE, HOX transcript antisense RNA (HOTAIR), lncRNA-small nucleolar RNA host gene 14 (SNHG14) or the circular RNA hsa_circ_0008309 [19,53,54,55]. Like in many solid tumors, the main factor to which expression could be relevant to predict patient outcome is CRNDE, the hsa-miR-136-5p sponge [19]. CRNDE was of peculiar interest in breast cancer since a recent meta-analysis confirmed this lncRNA could act as an oncogene which upregulated expression correlated with poor prognosis (late Tumor Node Metastasis grade) and tumor progression (high tumor size and lymph node metastasis) [56,57]. In vitro, CRNDE controls hsa-miR-136-5p level which, in turn, is able to target RASAL2 and Wnt/beta-catenin signaling in triple-negative breast cancer cells [18,19]. In the present study, we showed that hsa-miR-136-5p could modulate ERα36 expression independently of CRNDE. Thus, we added ERα36 as another piece to the puzzle of the complex epigenetic regulation of breast cancer progression. 

## 4. Materials and Methods

### 4.1. Reagents

4-hydroxytamoxifen (OHT), fulvestrant (ICI 182, 780), and 5-Aza-2′-deoxycytidine (decitabine, DAC) were purchased from Sigma-Aldrich, France. Stock solutions (10 mM) of each compound were prepared in dimethyl sulfoxide (DMSO) and further diluted in culture medium for in vitro cell treatment. All working solutions were freshly made just before treatment, and control cells were treated with DMSO, diluted with the same factor.

### 4.2. Plasmids

Reporter plasmids containing the miR-136-5p target sequence from ERα36 3’UTR downstream the luciferase open reading frame were constructed as follows: pMIR-Report-Luc-KLF4-FL vector was purchased from the nonprofit Addgene repository (Addgene plasmid #34597) as a gift from Michael Ruppert [58]. The KLF4 sequence was removed and replaced by either the wild-type ERα36 3’UTR sequence (5’-CCACCATCAAATCAAATTGA-3’) or the mutated one (5’-CCACCACTACAATACAATTGA-3’). The corresponding plasmids were renamed pMIR-Report-Luc-ERα36wt and pMIR-Report-Luc-ERα36mut, respectively. pEZX-PG04-Secreted Alkaline Phosphatase plasmid was purchased from GeneCoppoeia™ (Tebu-bio, Le Perray-en-Yvelines, France).

### 4.3. Cell Culture

MCF-7 and MDA-MB-231 cells were purchased in 2015 from ATCC^®^ (HTB-22™ and HTB-26™, respectively; Molsheim, France) and maintained in 10% fetal bovine serum (FBS) 1% Glutamine supplemented Dulbecco/Vogt modified Eagle’s minimal essential medium (DMEM) (GIBCO) or Roswell Park Memorial Institute medium (RPMI) (GIBCO) medium, respectively.

### 4.4. Transfection

Transient transfections of MCF-7 or MDA-MB-231 cells were performed using JetPRIME transfection reagent (Ozyme, Saint-Cyr-l’École, France) in accordance with the manufacturer’s instructions. Briefly, cells were plated at a density of 2.5 × 10^5^ cells per well in 6-well plates. After 24 h, transient transfection was performed with either 1 µg plasmid and/or 30 pmol (15 nM final) miRVana hsa-miR-136-5p mimic (5’-CAUCAUCAAAACAAAUGGAGUTT-3’) or inhibitor (5’-UCCAUCAUCAAAACAAAUGGAGU-3’) (Thermo Fischer, Illkirch, France) in serum-containing medium. The growth medium was renewed 4 hours after the transfection, and cells were treated with selected agents for 24 hours.

### 4.5. Luciferase/Alkaline Phosphatase Assays

The luciferase activity associated with the plasmid pMIR-Report-Luc-ERα36wt and the plasmid pMIR-Report-Luc-ERα36mut was assessed using the Bright-Glo™ luciferase assay system (Promega Corporation, Madison, WI, USA) in accordance with the manufacturer’s instructions. To normalize the results, pEZX-PG04-Secreted alkaline phosphatase plasmid was used, and the secreted alkaline phosphatase activity was detected on cell culture supernatant using the Secrete-Pair™ Gaussia luciferase dual and single luminescence assay kit (GeneCoppoeia™ Tebu-bio, Le Perray-en-Yvelines, France) in accordance with the manufacturer’s instructions. Luminescence was measured by a microplate reader (Victor x3, Perkin-Elmer, Villebon-sur-Yvette, France).

### 4.6. MicroRNA Extraction and Reverse Transcription

Micro and long RNAs were isolated using the mirVana™ miRNA Isolation Kit (Ambion, Austin, TX, USA) in accordance with the manufacturer’s instructions. The purity and quantity of RNA were assessed using the NanoDrop ND-2000C spectrophotometer (Thermo Scientific, Wilmington, DE, USA). The samples were used immediately or stored at −80 °C for future use. Reverse transcription (RT) and real-time PCR (qPCR) were carried out using TaqMan™ microRNA assay (Applied Biosystems, Foster City, CA, USA). RT reactions were performed in a volume of 15 μl, and each reaction contained 60 ng of microRNA. RT reactions were performed with a Mastercycler^®^ Gradient (Eppendorf, Montesson, France) with the following conditions: 16 °C for 30 min, 42 °C for 30 min, 85 °C for 5 min, and 4 °C on hold. Reactions without addition of reverse transcriptase (RT (−) controls) were performed alongside with cDNA synthesis of each sample and used in subsequent procedures to control the potential genomic DNA contamination. A total of 1.4 μl of RT reaction product was added in the 20 μl qPCR reaction mix containing TaqMan™ Universal PCR Master Mix II without UNG (Applied Biosystems). All TaqMan assays were run in triplicate on a CFX96 Touch™ real-time PCR detection system (Biorad, Marnes-la-Coquette, France). Real-time PCR cycling conditions consisted of 95 °C for 10 min, followed by 50 cycles of 95 °C for 15 s and 60 °C for 1 min. TaqMan Probe (hsa-miR-136-5p: 5’-UGAGCCCUCGGAGGACUCCAUUUGUUUUGAUGAUGGAUUCUUAUGCUCCAUCAUCGUCUCAAAUGAGUCUUCUGUGGGUUCU-3’; hsa-miR-423-5p: 5’-UGAGGGGCAGAGAGCGAGACUUUUCUAUUUUCCAAAAGCUCGGUCUGAGGCCCCUCAGU-3’). Assays were performed at least as independent experimental triplicates, and the mean values were used to calculate expression levels, using the ΔΔC(*t*) method referring to hsa-miR-423 housekeeping miR expression (as recommended by the manufacturer, Applied Biosystems).

### 4.7. Long RNAs RT and Real-time PCR Analysis

RT and real-time PCR analyses were performed as previously described [16]. The following primers were used for qRT-PCR: GAPDH forward (Fw) 5’-TGC-ACC-ACC-AAC-TGC-TTA-GC-3’, GAPDH reverse (Rev) 5’-GGC-ATG-GAC-TGT-GGT-CAT-GAG-3’, ERα36 forward (Fw) 5’-ATG-AAT-CTG-CAG-GGA-GAG-GA-3’, ERα36 reverse (Rev) 5’-GGC-TTT-AGA-CAC-GAG-GAA-ACC-3’, CRNDE forward (Fw) 5’-ATA-TTC-AGC-CGT-TGG-TCT-TTG-A-3’, and CRNDE reverse (Rev) 5’-ATA-TTC-AGC-CGT-TGG-TCT-TTG-A-3’. Assays were performed at least in triplicate, and the mean values were used to calculate expression levels, using the ΔΔC(*t*) method referring to GAPDH housekeeping gene expression.

### 4.8. Western Immunoblotting

Western blots were performed as described previously [10]. We used the primary antibody anti-ERα66 (F10, Santa Cruz Biotechnology, Heidelberg, Germany). The anti-α-Tubulin (GTX102079, Euromedex, Souffelweyersheim, France) was used as a loading control. Protein expression profiles were revealed with Clarity Western ECL Substrate (Biorad), and banding quantification was performed using the Image Lab 6.0.0 software (Biorad).

### 4.9. Patients and Samples

Cryopreserved samples (Biobank registration number AC-2008-174) from 17 patients treated at Cancerology Institute of Lorraine (Nancy, France) between 2014 and 2015 with histology proven T1 to T4 invasive breast cancer were used for this study. Among the 17 patients, 14 (82%) had an invasive ductal carcinoma and 3 (18%) had a nonspecific invasive carcinoma. Breast tumors were examined by hematoxylin and eosin staining and histopathology defined in accordance with the sixth edition of TNM [59]. Hormonal receptors, HER2 and Ki-67 expressions were assessed using immunohistochemistry (Dako, Les Ulis, France). Patients’ characteristics are detailed in Table S1. The investigations were carried out following the rules of the Declaration of Helsinki of 1975 (https://www.wma.net/what-we-do/medical-ethics/declaration-of-helsinki/), revised in 2013. All patients gave their informed consent for the use of their samples, and all data were anonymized prior to analysis to protect patient confidentiality. This study was performed within the framework of the tumor banking program of the Cancerology Institute of Lorraine including ethical considerations (approval AC-2013-1916 French Ministry of Research).

### 4.10. Bisulfite Sequencing

Bisulfite sequencing data of tumor samples were obtained from Active Motif’s Targeted Next-Gen Bisulfite Sequencing Service (Active Motif Europe, La Hulpe, Belgium).

### 4.11. DNA Methylation Analysis or ERα36 Promoter Sequence

Genomic DNA was harvested from MCF-10A, MCF-7 or MDA-MB-231, and bisulfite conversion of DNA was performed with the bisulfite conversion kit (Active Motif Europe, La Hulpe, Belgium) in accordance with the manufacturer’s instructions. Real-Time PCR analysis of methylated/unmethylated DNA was performed after conversion using specific primers (forward 5’-GAG-TTT-AAA-ATA-AGT-TAT-ATG-GAA-GTA-TAA-GTG-3’; Meth reverse 5’-CGA-CCG-CGC-TCC-TTC-CAC-AAT-AAC-TAC-GA-3’; Unmeth reverse 5’-CAA-CCA-CAC-TCC-TTC-CAC-AAT-AAC-TAC-AA-3’).

### 4.12. Statistical Analysis

Statistical analyses were performed with MATLAB R2018a software (MathWorks) using Student’s *t*-test for unpaired samples and ANOVA followed by Bonferroni post-hoc test for multigroup comparison, with a significant *p*-value threshold below 5%. * *p* < 0.05; ** *p* < 0.01; *** *p* < 0.001. Standard deviations or standard errors were indicated on figures as advocated by Altman and Bland [60].

## Supplementary Materials

Supplementary materials can be found at https://www.mdpi.com/1422-0067/20/11/2637/s1.
