# Supplementary material for: Dual Epigenetic Regulation of ERα36 Expression in Breast Cancer Cells
Source: Int J Mol Sci. 2019 May 29;20(11):2637. doi: 10.3390/ijms20112637 (PMC6600239; doi:10.3390/ijms20112637)
Supplement: Supplementary file 1 [file ijms-20-02637-s001.pdf]

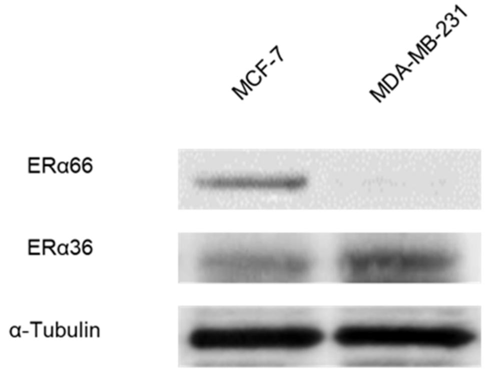

Figure S1: Representative image of MCF-7 or MDA-MB-231 cells submitted to western blotting as described previously [10]. We used the primary antibodies anti-ERα66 (anti-ERα-F10, Santa Cruz Biotechnology) and anti-ERα36 (anti-ERα-G20, Santa Cruz Biotechnology). The anti-α-Tubulin (GTX102079, Genetex) was used as a loading control. Protein expression profiles were revealed with Clarity Western ECL Substrate (Biorad).

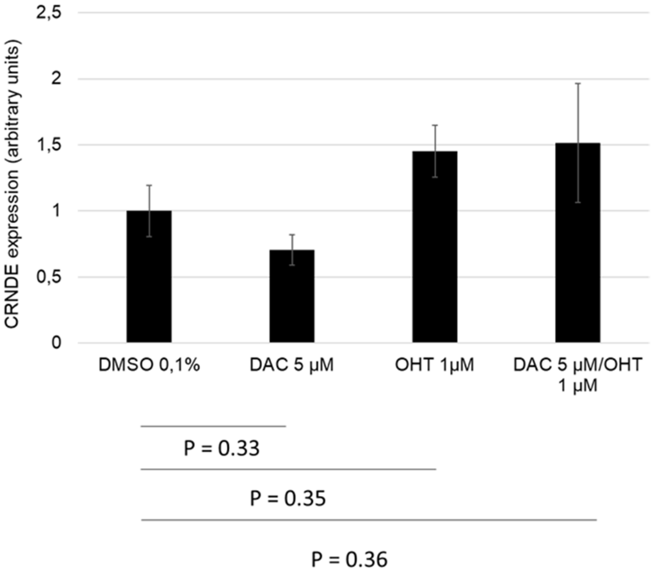

Figure S2: Expression of CRNDE after 48h treatment with DAC, OHT or DAC+OHT as measured by RT-qPCR.

MCF-7 cells were treated for 48h with DMSO 0.1% (as control), DAC 5 µM, OHT 1µM or by a combination OHT 1µM/DAC 5µM. CRNDE expression was measured by RT-qPCR analysis. Each bar represents mean ± S.E.M. N ≥ 3. *: *P* <0.05

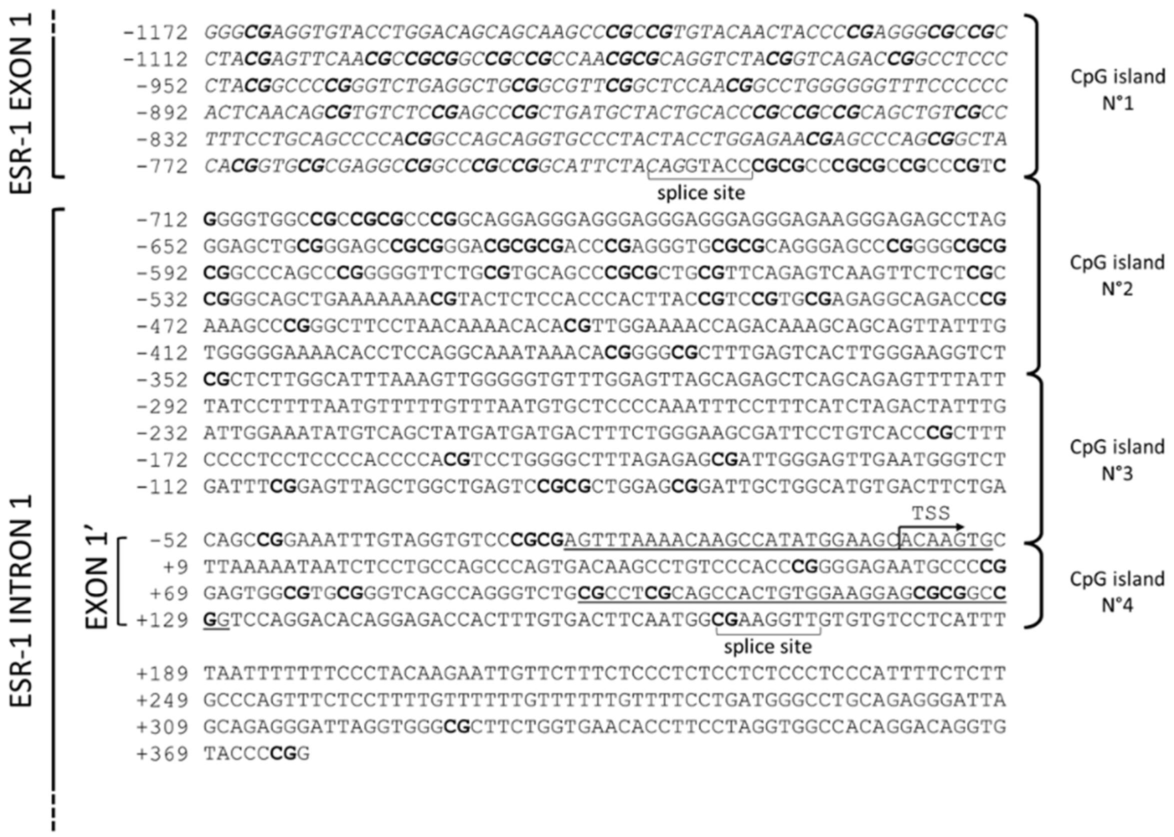

Figure S3. Exon1/intron1 part of ESR1 genomic sequence.

(>gi|224589818|ref|NC_000006.11|:152129448-152130362 Homo sapiens chromosome 6, GRCh37.p10 Primary Assembly). Exon1 sequence is italicized and refers to the canonical ERα66 transcribed sequence while exon 1’ refers to the variant ERα36. Nucleotides are numbered from ERα36 transcription start site (TSS, arrow). Exon1/intron1 and exon1’/intron1 splice sites are annotated and CpG dinucleotides are in bold. The use of EMBOSS CpG plot (http://www.ebi.ac.uk/Tools/seqstats/emboss_cpgplot/) allowed to design four ESR1 genomic regions as CpG islands on both sides of (i) the ESR-1 exon1/intron1 boundary (CpG islands No.1 and 2, respectively) and (ii) the ERα36 transcription start site (CpG islands No.3 and 4). PCR primers used for CpG island amplification after bisulfite conversion are underlined.
